# Beyond polio: advanced human *in vitro* models for studying non-polio enterovirus pathogenesis

**DOI:** 10.1128/jvi.01699-25

**Published:** 2026-09-30

**Authors:** Ashley Kelly Pereirinha da Silva, Bjoern Meyer, Lisa Bauer

**Affiliations:** 1 Department of Viroscience, Erasmus University Medical Center 686104, Rotterdam, The Netherlands; 2 Medical Faculty, Institute of Medical Microbiology and Hospital Hygiene, Otto-von-Guericke-University Magdeburg 661301, Magdeburg, Germany; Universiteit Gent, Merelbeke, Belgium

**Keywords:** non-polio enteroviruses, organoids, stem cells, pathogenesis

## Abstract

While efforts to eradicate poliovirus are ongoing, non-polio enteroviruses (NPEV) continue to pose a substantial public health threat. NPEVs cause a wide range of diseases, from mild self-limiting infections to severe respiratory, neurological, and cardiac complications, with the highest burden occurring in children and immunocompromised populations. Recent advances in human stem cell-derived cultures, organoids, air-liquid interface systems, and assembloids have transformed the study of NPEV pathogenesis by providing physiologically relevant models that recapitulate human tissue architecture, cellular complexity, and host responses. In this review, we discuss how these advanced *in vitro* systems have enhanced our understanding of the determinants of viral tropism, including receptor usage, epithelial polarity, tissue-specific susceptibility, and viral genetic variation. We further highlight insights into serotype-dependent differences in innate immune responses, tissue injury, and dissemination mechanisms. Finally, we explore the potential of these models for antiviral discovery and personalized therapeutic development, emphasizing their value for advancing both fundamental and translational NPEV research.

## INTRODUCTION

The genus *Enterovirus*, within the family *Picornaviridae*, comprises 15 species, of which the *Enterovirus alphacoxsackie*, *betacoxsackie*, *coxsackiepol*, and *deconjuncti* and *Rhinovirus alpharhino*, *betarhino,* and *cerhino* harbor many medically important pathogens, which are among the most common infections in mankind (1, 2). Rhinoviruses, polioviruses, and over 100 non-polio enterovirus (NPEV) serotypes (e.g., enteroviruses [EV], echoviruses [E], and coxsackievirus [CV]) cause a large spectrum of diseases, ranging from mild and self-limiting to severe and potentially fatal (3). Poliovirus, the causative agent of poliomyelitis, was the first known EV, with 1950s epidemics driving the development of oral and inactivated vaccines and universal childhood immunization programs (4). The Global Polio Eradication Initiative, launched in 1988, has nearly eradicated wild-type poliovirus with only small endemic areas remaining in Afghanistan and Pakistan (5, 6). Global surveillance revealed frequent detection of NPEVs in environmental and clinical samples, demonstrating that even in an almost polio-free world, NPEVs can cause devastating diseases such as acute flaccid paralysis (AFP), myocarditis, pneumonia, and sometimes even fatalities (7–10). The re-emergence of a poliomyelitis-like neurological disease, particularly associated with EV-A71, EV-D68, various echoviruses, and CVA viruses, raised significant concerns regarding the neurotropic potential of NPEVs (11–13). Despite their substantial global burden, the molecular mechanisms driving diseases remain poorly understood. This limited understanding hampers the development of antivirals and therapeutic interventions. Increasing awareness of NPEVs and advancing our understanding of their pathogenesis are critical to improve clinical management and preparedness for future outbreaks. In this review, we examine the spectrum of NPEV diseases, highlighting how advanced cell models shed light on pathogenesis and therapeutic strategies.

## TRANSMISSION AND CLINICAL FEATURES

**Fig 1:**
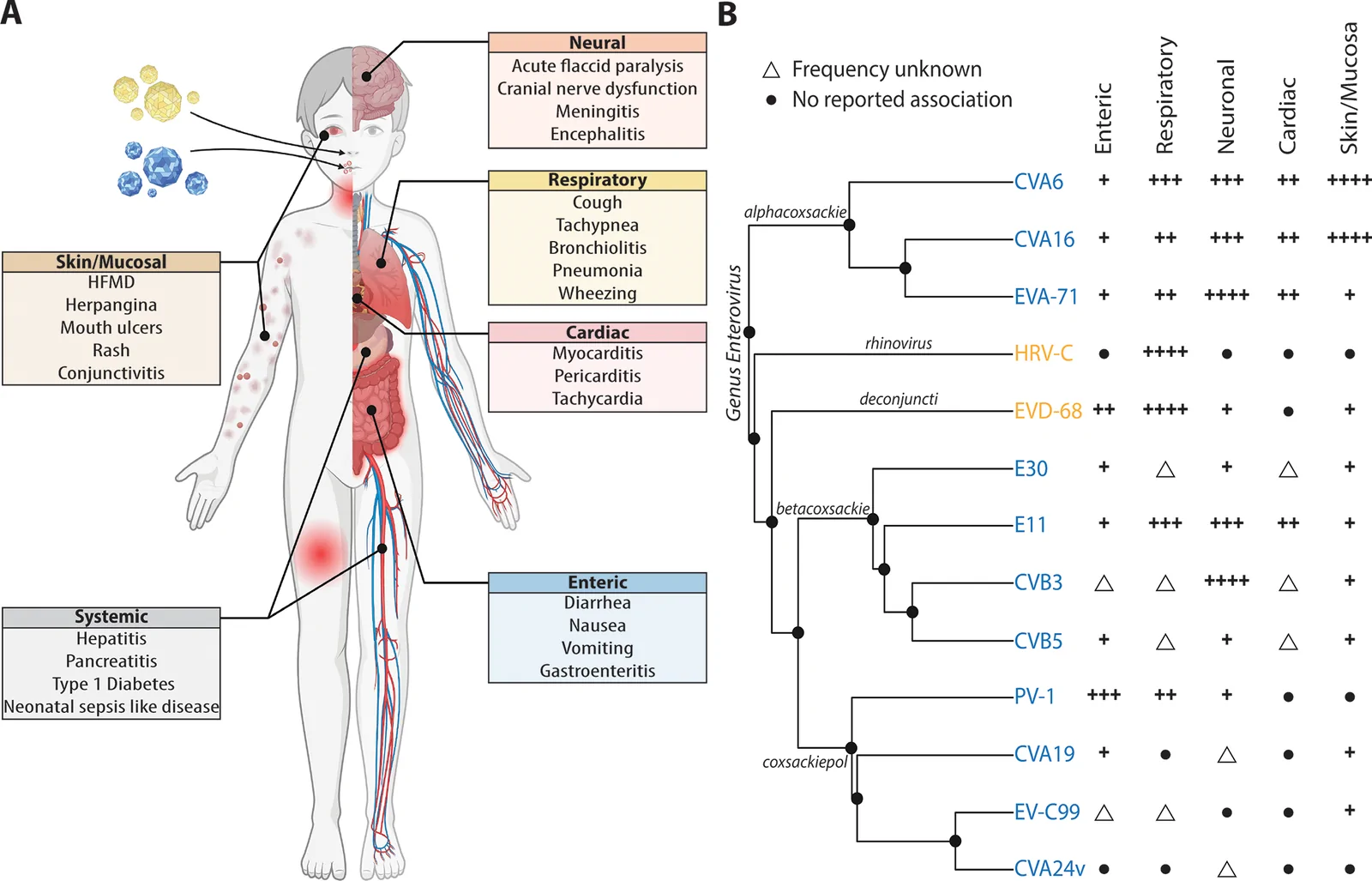
Presentation of clinical manifestations and frequency of NPEVs. (**A**) Overview of prevalent clinical manifestations throughout the body. (**B**) Frequency of clinical manifestations by organ system. Phylogenetic tree of EVs was generated with Clustal Omega (91) based on the structural protein VP1 of prototypic strains as indicated at https://www.picornaviridae.com. Symbols denote the relative frequency with which each manifestation has been reported in hospitalized patients in the literature for the corresponding virus: +low frequency, ++moderate frequency, +++high frequency, ++++very high frequency. Relative frequencies were derived from the available surveillance literature. For each virus, the proportion of cases attributed to each disease outcome was estimated from reported case counts to enable comparison across studies with differing sample sizes and reporting formats. The highest reported proportion across available studies was used to represent each group. Exact percentages are not reported in the figure, as the underlying surveillance data are incomplete and heterogeneous across sources, and precise values could imply a level of accuracy that is not supported by the available evidence. △ indicates that the manifestation has been described, but its frequency could not be determined from available data. • indicates no reported association between the virus and that organ system. Associated references are listed in Table 1. Viruses that are primarily transmitted via the fecal-oral route are highlighted in blue, and viruses transmitted through the respiratory route are highlighted in yellow.

Most NPEVs are transmitted through the fecal–oral route, while EV-D68 is transmitted via the respiratory route. Following exposure, primary replication occurs in the epithelium of the mucosal surfaces of the gastrointestinal (GI) or upper respiratory tract (RT) (14). NPEVs are ubiquitous in early childhood and typically asymptomatic, contributing to substantial underdetection. Although most infections are self-limiting, chronic or severe conditions and fatalities can occur, particularly among infants, children, and immunocompromised patients (15, 16). Disease severity is likely determined by an interplay of virus serotypes, host-related factors, and socioeconomic status. Furthermore, a comprehensive understanding of the true incidence, disease burden, and clinical spectrum of NPEV infections remains challenging due to inadequate surveillance, limited use of EV serotyping in routine clinical practice, and difficulty distinguishing causal relationships from incidental associations (17). The potential emergence of more pathogenic strains over time adds further complexity. For example, although EVs have been associated with bronchiolitis or other diseases, serotype data are often unavailable, limiting attribution to specific NPEV serotypes (18, 19). Table 1 summarizes reported clinical diagnoses by NPEV serotype, showing that manifestations vary by serotype and tissue tropism. Despite these limitations, available evidence indicates that NPEVs can cause a broad spectrum of clinical manifestations, reflecting differences in viral serotype, tissue tropism, and disease pathogenesis. The initial site of infection often shapes the earliest clinical presentation. GI-tract infection may result in abdominal pain, vomiting, nausea, or diarrhea (Fig. 1, Table 1). However, it is noteworthy that the GI-symptoms associated with NPEV infections are not as well recognized as with noroviruses and rotaviruses (20, 21). NPEVs, including EV-D68, CVA4, CVA6, CVA10, EV-A71, and E11, can cause respiratory diseases ranging from mild upper RT symptoms, such as rhinorrhea, sneezing, sore throat, and productive cough, to severe lower RT involvement (Fig. 1). EV-D68 is particularly associated with wheezing, exacerbation of asthma, pleural effusion, pulmonary consolidation, and pneumonia, sometimes requiring hospitalization (Table 1) (22).

**TABLE 1:** Clinical manifestations associated with NPEVs^*a*^

Anatomical location	Diagnosis/disease	Alphacoxsackie	Betacoxsackie	Coxsackiepol	Deconjuncti	Ref
Enteric	Gastroenteritis	CVA2, CVA4, CVA5, CVA6, CVA8, CVA10, CVA12, CVA14, CVA19, EV-A71	CVA9, CVB1–CVB6, E1, E6, E11, E12, E14, E18, E25, E30	CVA1, CVA11, CVA13, CVA17, CVA19–CVA22, EV-C99	EV-D68	(23–28)
	Unspecified gastrointestinal disorders^*b*^	CVA2, CVA4, CVA5, CVA6, CVA8, CVA10, CVA14, CVA16, EV-A71	CVA9, CVA24 CVB1–CVB6, E1-E7, E9, E11–E15, E18–E21, E24–E27, E30, E32, E33, EV-B75, EV-B77, EV-B80, EV-B83, EV-B84, EV-B93	CVA1, CVA13, CVA17, CVA19, CVA21, CVA22, CVA24, EV-C99, EV-C105	EV-D68	(23, 25, 29, 30)
Respiratory	Influenza-like illnesses	CVA2, CVA4, CVA5, CVA6, CVA7, CVA8, CVA10, CVA12, CVA14, CVA16, EV-A71	E1–E7, E9, E11, E16–E21, E25, E30, E32, CVA9, CVB1–CVB6	CVA20, CVA21, CVA24, EV-C105, EV-C117	EV-D68	(23, 24, 27, 31–34)
	Rhinitis^*c*^				EV-D68	(35)
	Pharyngitis^*c*^	CVA10			EV-D68	(35, 36)
	Tonsillitis^*c*^	CVA6				(37)
	Bronchitis^*c*^	CVA6			EV-D68	(33, 37, 38)
	Bronchiolitis^*c*^				EV-D68	(39)
	Pneumonia	CVA6	CVB4, CVB5	CVA21, CVA24, EV-C104, EV-C118	EV-D68	(33, 37, 38, 40, 41)
	Pneumomediastinum^*c*^				EV-D68	(39)
Neurological	Acute flaccid paralysis/ acute flaccid myelitis	CVA2–CVA8 CVA10, CVA12, CVA14, CVA16, EV-A71, EV-A76, EV-A90	CVA9, CVB1–CVB6, E1–E7, E9, E11–E22, E24–E27, E29–E33, EV-B73–EV-B75, EV-B77, EV-B79–EV-B81, EV-B83, EV-B85–EV-B88, EV-B93, EV-B97, EV-B106, EV-107	CVA1, CVA11, CVA13, CVA17, CVA20, CVA21, CVA22, CVA24, EV-C96, EV-C99, EV-C105, EV-C109	EV-D68, EV-D70, EV-D94	(16, 26, 42)
	Encephalitis	CVA2–CVA8, CVA16, EV-A71	CVA9, CVB1, CVB2, CVB3, CVB4, CVB5, E3–E7, E9, E11, E13, E14, E16–E20, E24, E25, E27, E30, E33	CVA11, CVA13	EV-D68, EV-D70	(23, 24, 43)
	Brainstem/ rhombencephalitis	CVA16, EV-A71	CVA9, CVB2, CVB4		EV-D68	(23, 27, 42, 44, 45)
	Meningitis	CVA2–CVA8, CVA10, CVA14, CVA16, EV-A71	CVA9, CVB1–CVB6, E1–E7, E9, E11–E21, E24, E25, E27, E29, E30, E31–E33, EV-B74	CVA11, CVA13, CVA17, CVA19, CVA22, CVA24, EV-C105, EV-C109	EV-D68, EV-D70	(16, 23, 24)
	Acute polyradiculoneuritis^*c*^	CVA4				(46)
	Cranial nerve radiculitis^*c*^	EV-A71				(27)
Cardiac	Pericarditis	CVA4, CVA16, EV-A71	CVB1–CVB6, E1, E3, E6, E7, E9, E11, E13, E16,		EV-D68	(26, 45)
	Myopericarditis	CVA16, EV-A71	CVA9, CVB1–CVB6, E1–E3, E6, E7, E9, E11, E13, E16, E30			(24)
	Myocarditis	CVA2, CVA4–CVA8, CVA10, CVA16, EV-A71	E9, E11, CVB1–CVB6	CVA1	EV-D68	(23, 27, 43, 45)
Dermatological	HFMD	CVA2, CVA4–CVA8, CVA10, CVA12–CVA14, CVA16, CVA19, EV-A71	CVA9, CVB1–CVB5, E3, E4, E6, E7, E9, E11, E15, E16, E18, E19, E30, EV-B84	CVA1, CVA13, CVA19–CVA22, CVA24, EV-C99		(23, 25, 37, 43)
Oral mucosa	Herpangina	CVA2–CVA6, CVA8, CVA10, CVA16, EV-A71	CVA9, CVB1–CVB6, E3, E6, E9, E11, E16, E25, E30	CVA1, CVA20, CVA21, CVA22, CVA24		(26, 42)
Conjunctiva	Acute hemorrhagic conjunctivitis	CVA6		CVA24v	EV-D70	(47)
Others	Lymphadenopathy^*c*^	CVA6	CVB5, E7			(48–50)
	Hepatitis		CVA9, CVB1–CVB5, E1, E3, E6, E7, E9, E11, E14, E17, E19, E20, E25, E30	CVA20, CVA21, CVA24		(24, 26)
	Pancreatitis	CVA4, CVA16, EVA71	CVB1–CVB6, E6, E7, E11, E25, E10, E30			(24, 26)
	Type 1 diabetes		CVA9, CVB1-CVB6, E3-E6, E11, E16, E18, E24, E25, E30	CVA21, CVA24	EV-D70	(24)

^*a*^ The table lists clinical diagnoses or diseases in which each EV genotype has been reported as cause of disease. Despite extensive literature search, we cannot exclude the possibility that relevant serotypes were missed and are, therefore, not included in this table.

^*b*^ Gastrointestinal disorders presenting with symptoms such as vomiting, nausea, diarrhea.

^*c*^ Most case reports and surveillance data do not genotype the serotypes. Disease associations should be interpreted with caution.

After initial replication at the primary site of infection, NPEVs can spread to regional lymph nodes which precedes the onset of viremia. Echoviruses and CVBs are one of the most common causes of viral sepsis in neonates (51). The broad secondary tissue tropism varies between serotypes due to their utilization of a wide variety of distinct receptors (Table 2). For example, different echoviruses bind to the complement decay-accelerating factor (DAF), while CVBs interact with coxsackievirus and adenovirus receptor (CAR) (52, 53). In contrast, viruses can utilize pleiotropic receptors exemplified by EV-D68 that binds to ICAM-5, MFSD6, and glycans (54–59). The diverse tissue distribution of receptors partially explains the broad organ tropism and the variation in clinical manifestations. The greatest clinical relevance of NPEVs arises from their neurotropic potential being responsible for a large proportion of encephalitis, meningitis, and AFP (60). Echoviruses and CVB serotypes can affect the cardiovascular system causing pericarditis, myocarditis, myopericarditis, dilated cardiomyopathy, and very rarely acute heart failure (Fig. 1; Table 1). NPEVs can infect skin, mucosal, and subconjunctival membranes, resulting in rashes, hand-foot-and-mouth disease (HFMD), herpangina, and acute hemorrhagic conjunctivitis (3, 61, 62) (Fig. 1; Table 1). Other organ systems that can be affected by NPEVs include the liver and pancreas, leading to hepatitis and pancreatitis (10, 63) (Fig. 1). NPEVs have also been associated with the development of type 1 diabetes although this link remains under debate (64).

**TABLE 2:** Entry factors of NPEVs^*a*^

Entry factor	Virus	Entry factor expression (from highest to lowest)
SCARB2 (65, 66)	EV-A71, CVA7, CVA14, CVA16	Cerebral cortex, cerebellum, hippocampus, caudate, thyroid gland, adrenal gland, nasopharynx, bronchus, lung, salivary gland, stomach, duodenum, small intestine, colon, rectum, liver, gallbladder, pancreas, urinary bladder, testis, epididymis, seminal vesicle, prostate, fallopian tube, endometrium, placenta, heart muscle, skin, appendix, spleen > kidney, ovary, smooth muscle, adipose tissue, lymph node, tonsil > oral mucosa, esophagus, cervix, skeletal muscle, soft tissue, bone marrow
CAR (*CXADR*) (53)	CVB1, CVB2, CVB3, CVB4, CVB5, CVB6	Lung, stomach, duodenum, gallbladder, kidney, urinary bladder, prostate, skin > cerebral cortex, thyroid gland, adrenal gland, nasopharynx, bronchus, small intestine, colon, rectum, liver, pancreas, testis, seminal vesicle, vagina, endometrium, cervix, appendix, tonsil, bone marrow > cerebellum, hippocampus, caudate, parathyroid gland, oral mucosa, salivary gland, esophagus, epididymis, ovary, fallopian tube, placenta, breast, heart muscle, skeletal muscle, soft tissue, adipose tissue, spleen, lymph node, smooth muscle
ICAM5 (55)	EV-D68	Cerebral cortex > hippocampus, caudate, placenta
ICAM1 (67)	CVA21, CVA24	Lung, kidney, endometrium > testis, appendix, spleen, lymph node, tonsil, bone marrow > urinary bladder, fallopian tube, colon > cerebral cortex, skin
PSGL-1 (CD162) (68, 69)	EV-A71, CVA2, CVA7, CVA10, CVA14, CVA16	Cerebellum, hippocampus, caudate, lymph node > cerebral cortex, adrenal gland, lung, pancreas, kidney, spleen, tonsil > breast, bone marrow
Annexin II (ANXA2R) (70, 71)	EV-A71	No data available
DC-SIGN (CD209) (72)	EV-A71	Placenta > lung, testis, bone marrow
Nucleolin (NCL) (73)	EV-A71	Cerebral cortex, cerebellum, hippocampus, caudate, thyroid gland, parathyroid gland, adrenal gland, nasopharynx, bronchus, lung, oral mucosa, salivary gland, esophagus, stomach, duodenum, small intestine, colon, rectum, liver, gallbladder, pancreas, kidney, urinary bladder, testis, epididymis, seminal vesicle, prostate, vagina, ovary, fallopian tube, endometrium, cervix, placenta, breast, heart muscle, smooth muscle, skeletal muscle, soft tissue, adipose tissue, skin, appendix, spleen, lymph node, tonsil, bone marrow
Vimentin (74)	EV-A71	Retina, thyroid gland, lung, kidney, testis, epididymis, seminal vesicle, ovary, fallopian tube, placenta, breast, soft tissue, adipose tissue, skin, appendix, spleen, lymph node, tonsil, bone marrow > caudate, pancreas, endometrium, cervix > cerebral cortex, hippocampus, adrenal gland, nasopharynx, bronchus, liver, vagina, colon, rectum
DAF (CD55) (52, 75, 76)	CVA21, CVB1, CVB3, CVB5, E3, E6, E7, E11, E12, E13, E19, E20, E21, E25, E29, E30	Lung, testis, ovary, placenta, skin > cerebral cortex, cerebellum, adrenal gland, salivary gland, kidney > nasopharynx, bronchus, stomach, duodenum, small intestine, colon, rectum, urinary bladder, vagina, endometrium, cervix, appendix, tonsil, bone marrow > soft tissue
Integrin α_v_β_3_(CD61) (77)	CVA9, E1, E9	Bone marrow
Integrin α_2_β_1_ (CD49B, VLA-2) (78)	E1, E8	Adrenal gland, nasopharynx, bronchus, stomach, duodenum, small intestine, colon, rectum, kidney, urinary bladder, epididymis, seminal vesicle, endometrium, breast, skin, appendix, tonsil > caudate, parathyroid gland, lung, oral mucosa, salivary gland, esophagus, liver, gallbladder, testis, vagina, ovary, fallopian tube, cervix, spleen, bone marrow > cerebral cortex, thyroid gland, pancreas, prostate, placenta, heart muscle, skeletal muscle, cerebellum, hippocampus, smooth muscle, soft tissue, adipose tissue, lymph node
MFSD6 (54, 57, 79)	EV-D68	Cerebellum, thyroid gland, bronchus, stomach, small intestine, colon, rectum, gallbladder, pancreas, epididymis, fallopian tube, placenta > cerebral cortex, adrenal gland, lung, oral mucosa, salivary gland, duodenum, liver, kidney, urinary bladder, testis, seminal vesicle, prostate, ovary, endometrium, cervix, skeletal muscle, appendix, lymph node, tonsil, bone marrow > hippocampus, caudate, esophagus, vagina, breast, heart muscle, smooth muscle, soft tissue, adipose tissue, skin, spleen
Kremen1 (80)	CVA2, CVA3, CVA4, CVA5, CVA6, CVA10	No data available
FcRn (neonatal fc-receptor) (81, 82)	CVA9, E1–E3, E6, E7, E9, E11, E13–E15, E18, E25, E26, E30 EV-B25	Cerebellum > cerebral cortex, hippocampus, caudate, lung, testis, heart muscle
Sulfated glycosaminoglycan (sGAG) (83–86)	CV-A9, CV-B3, E5, E6, EV-A71, EV-D68	In adult mice (87): cartilage > spleen > uterus > kidney > skin > bladder > stomach > colon > adrenal gland > lung > eye > brain > salivary gland > intestine > lymph node > heart > thymus > ovary > testicle > muscle > seminal gland > liver > pancreas > harderian gland
Sialic acid (SIA) (56, 58, 88, 89)	CVA24v, EV-D68, EV-D70, EV-D111	N-glycans in humans (90): ovary > adrenal gland > fat > brain > esophagus > intestine, testis > liver > prostate > kidney, colon > urinary bladder, muscle > pancreas, spleen, lung > stomach > heart; O-glycans: no data obtained

^*a*^ Overview of entry receptor protein expression levels in tissues was retrieved from the human protein atlas (https://www.proteinatlas.org/). Data on sGAGs and SIA were obtained from studies investigating and comparing glycan display patterns across tissues in the same study.

## ANIMAL MODELS TO STUDY NON-POLIO ENTEROVIRUS PATHOGENESIS

Studying NPEV pathogenesis in human biopsies or postmortem tissues offers valuable insights into disease progression and tissue-specific pathology. However, the limited access of such specimen makes the use of appropriate animal models essential for uncovering the pathological mechanisms. Developing suitable models is challenging because NPEVs are human viruses that generally replicate inefficiently in other species. In addition, NPEVs are associated with a variety of clinical manifestations involving multiple organ systems, making it difficult to establish animal models that accurately recapitulate the complexity of multi-organ human disease.

A variety of animal models such as non-human primates, rodents (e.g., mice, cotton rats, tree shrews, and guinea pigs), and ferrets have been established to study NPEV pathogenesis (92–94). However, most of them only recapitulate certain pathological aspects of infection, while having significant constraints. For instance, experimental infection of pigtailed macaques, African green monkeys, cynomolgus macaques, and rhesus macaques typically show asymptomatic or mild disease, consistent with the clinical presentation of most human NPEV infections. However, these models do not recapitulate the neurological manifestations that occur in a subset of patients (92, 93, 95, 96). Mouse models are widely used because of their accessibility and cost-effectiveness. However, experimental NPEV infections frequently require the use of mouse-adapted virus strains (97), transgenic animals expressing human receptors (98–100), neonatal mice (101–103), or immunocompromised animals (104, 105). These approaches facilitate infection, but they can alter viral tropism, disease progression, and host-pathogen interaction. Consequently, these models often fail to accurately reflect human receptor usage, immune maturation, and host immune responses, thereby limiting their value in studying pathogenesis.

Although most animal models fail to fully recapitulate the spectrum of NPEVs-associated pathology observed in humans, including lesions in the GI-tract (106), RT (101, 107, 108), liver (100), skin and mucosal surfaces (109, 110), or central nervous system (CNS), they have been particularly useful to model cardiac disease (e.g., myocarditis, pericarditis) and pancreatitis induced by CVB (111, 112). The limitations of available models are apparent when studying respiratory and neurological disease. The guinea pig model has been used to study human respiratory virus infections, but EV-D68 fails to cause the lesions (113). Cotton rats develop pulmonary lesions following EV-D68 exposure, demonstrating some capacity to model respiratory disease (107); however, their restricted susceptibility to other NPEV isolates, absence of paralytic manifestations, and limited applicability for longitudinal disease progression studies reduce the model’s translational relevance. Ferrets similarly fail to develop characteristic respiratory signs, such as sneezing, coughing, nasal discharge, or respiratory distress, nor do they exhibit paralysis or other neurological complications (108, 114). Neurological disease has also proven difficult to model accurately. AG129 mice, which lack type-I and type-II-interferon receptors, have been used to demonstrate strain-dependent differences in EV-A71 neuropathogenesis. However, these studies relied on mouse-adapted viral strains and intraperitoneal inoculation, both of which diverge substantially from natural human infection and may influence disease outcome (115).

Importantly, although animal models do not always fully recapitulate pathological aspects, this does not diminish their overall utility. Rather, their suitability depends on the specific research question being addressed, and several models remain highly informative for evaluating the efficacy of therapeutic interventions. To fully recapitulate the human disease caused by NPEVs, it requires complementary studies that use human samples, *in vivo* animal models, and *in vitro* approaches. The remaining challenge at present is identifying which findings in the various models can be extrapolated to the pathogenesis in humans and which must be carefully weighed against suboptimal pathogenesis models.

## *IN VITRO* MODELS TO STUDY NON-POLIO ENTEROVIRUS PATHOGENESIS

Virus characterization and pathogenesis used to be studied in cancerous or immortalized cell lines. Although Vero, HEK293T, RD, HeLa, A549, and SK-N-SH cells support NPEV replication, their simplified and homogeneous nature limits the assessment of cell type-specific tropism and innate immune responses. In addition, these cell lines frequently exhibit altered interferon (IFN) signaling, which may affect viral replication, thereby limiting their translational relevance.

**Fig 2:**
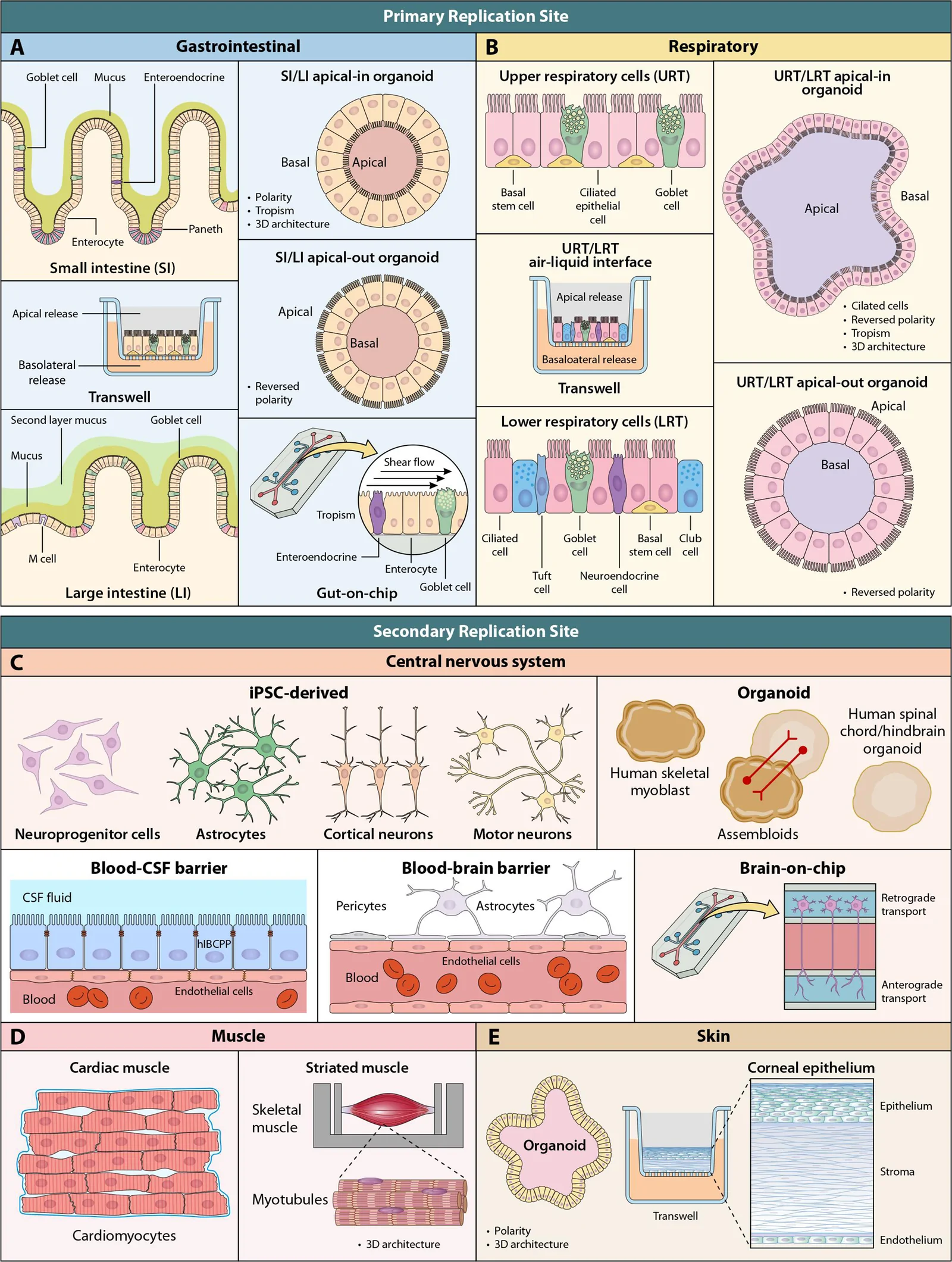
Advanced *in vitro* cell models to study NPEV infection across human tissues. Advanced cell models of (**A**) gastrointestinal (small and large intestine), (**B**) the lungs (lower and upper tracts; currently, no studies have used URT organoids to investigate NPEV infection), (**C**) central nervous system (brain and spinal cord), (**D**) muscle (cardiac and striated), and (**E**) skin, which are used in NPEV research, are illustrated. These models comprise hPSC-derived cells, co-culture models, organoids, ALI respiratory cultures, transwell insert systems, and organ-on-a-chip/microfluidic platforms. These systems enable mechanistic investigation of EV cell tropism, epithelial polarity, barrier integrity, and virus–host interactions.

Human stem cell- and organoid-based systems have revolutionized the cell culture landscape by enabling physiologically relevant disease modeling. Human pluripotent stem cells (hPSC, referring to embryonic and inducible PSC)-derived models rely on the direct differentiation of stem cells into distinct cell types representative of various tissues (116). Organoids are cell-derived 3D structures that self-assemble to organ-like complexity and preserve multicellular diversity (117) (Fig. 2). Donor tissue-derived organoids preserve patient-specific phenotypes such as age, sex, genetic background, and pre-existing conditions (e.g., asthma or COPD) (118, 119), making them particularly valuable for personalized medicine (120). These models have become widely used tools for studying viral disease pathogenesis, host susceptibility, inter-individual variation in infection outcome, and therapeutic testing (121). The organoid toolbox is continuously expanding and improving by incorporating different cells (e.g., tissue-resident and circulating immune cells) (122) and developing specialized configurations to address specific research questions, including multicellular transwell-based systems, apical-in and apical-out orientations, and the fusion of distinct organoids into increasingly complex systems called assembloids (Fig. 2). Transwell systems separate the apical and basolateral compartments, allowing investigation of polarized processes such as NPEV entry, release, and barrier crossing. Whereas conventional organoids are typically apical-in, apical-out organoids expose the luminal surface to the external environment, facilitating studies of viral attachment and entry. Air–liquid interface (ALI) cultures promote epithelial differentiation, mucus production, and barrier formation, particularly in respiratory and intestinal models.

## ENTERIC SYSTEM

Most NPEVs are transmitted via the fecal-oral route and initiate infection within the GI-tract. Despite the central role of the gut in NPEV transmission and pathogenesis, very little is known about the initial pathology, viral replication, cellular tropism, and dissemination.

### Intestinal cell tropism

The GI-tract is not a uniform environment but consists of distinct regional morphologies creating diverse environments influencing NPEV infection. The small intestine features villi lined by polarized epithelial cells, including enterocytes, goblet cells, enteroendocrine cells, and Paneth cells, whereas the large intestine presents a flatter surface with higher goblet cell density, producing a thick mucus layer (123) (Fig. 2A). Enteroids and colonoids, derived from the small intestine and colon, represent the diversity of intestinal epithelial cells, tissue organization, and essential gut functions, which provide valuable insight into the cellular tropism within the intestinal epithelium. Enterocytes have been identified as the predominant target cells for various NPEVs, including CVA16, EV-A71, CVB, E11, CVA13, CVA20, and EV-C99 (124–126). In addition, enteroendocrine cell support infection by E11 and EV-C99 (124, 125).

### Determinants of intestinal tropism

Multiple entry factors, including SCARB2, PSGL-1, vimentin, Annexin II, and glycans, can be utilized by EV-A71 (Table 2). Those expression levels are thought to define the cellular tropism (65, 68, 70, 74, 127). However, receptor expression alone does not predict tropism; rather, it depends on the experimental model and intestinal region. In transwell cultures derived from fetal intestinal crypts, goblet cells showed the highest SCARB2 expression and are preferentially infected by EV-A71 despite representing a minority cell population (128). In contrast, in well-differentiated polarized apical-out colonoids, SCARB2 is predominantly expressed on the apical surface of enterocytes coinciding with EV-A71 antigen detection following infection (129). In EV-A71 infected enteroids, also enterocytes are the primary target cell. Whether the SCARB2 contributed to this was not elucidated (126). Most studies evaluate SCARB2 and sulfated glycosaminoglycans (sGAGs) as entry receptors of EV-A71. The distribution of other reported receptors, such as PSGL-1, vimentin, Annexin II, and glycans, has not been systematically explored within intestinal organoid systems. Together, these findings suggest that cellular tropism cannot be explained solely by receptor expression. Rather, EV-A71 infection likely reflects the combined influence of multiple entry factors, including epithelial differentiation status and regional intestinal identity; a principle shared by other NPEVs. This gap highlights the need to systematically assess receptor expression profiles across intestinal cell types since expression of multiple entry factors across models and cell types likely underlies differences in NPEVs' tropism.

Beyond receptor expression, cell polarity also determines NPEV infection. Comparative studies of E11 and EV-A71 in intestinal epithelium on transwells demonstrated distinct patterns of polarized infection. EV-A71 prefers to infect goblet cells from the apical side, whereas E11 showed a preference for infection of enterocytes and enteroendocrine cells via the basolateral side (128). Differences in infection polarity have also been observed between EV-A71 isolates in a 2D human fetal intestinal model. EV-A71 C1-480-Q was able to infect the monolayers from both compartments, while EV-A71 C1-1185-E infected from the basolateral compartment (127). The factors governing infection polarity remain poorly understood. Characterizing these differences will aid insight into viral tropism that may influence both viral shedding and systemic dissemination.

### Epithelial integrity and cell death

A consistent finding across intestinal organoid models is that NPEV infection triggers serotype-specific forms of morphological alterations and cell death (124, 130). In fetal enteroids, E11 induces necrosis-like cell death, leading to loss of crypt morphology, impaired barrier integrity, and extensive tissue destruction, whereas similar effects were not observed following EV-A71 or CVB3 infection (124). In small intestinal organoids, EV-A71 and CVB2 induced cell death, while the respiratory-transmitted EV-D68 did not (130). Despite inducing comparable levels of cell death, NPEV serotypes differ in their effect on organoid structural integrity, suggesting different pathogenic mechanisms (130). Cell differentiation status influences the cellular death response to infection. In the intestinal epithelial-like cell line C2BBe1, EV-A71 infection induces apoptosis in undifferentiated cells but not in differentiated ones (131). In a Gut-on-a-Chip model (Fig. 2A) using Caco-2 cells exposed to fluid flow and peristalsis-like mechanical movement, CVB1 was preferentially released apically and induced apoptosis, while shear stress and villus morphology loss further amplified infection (132). Loss of epithelial integrity, villus destruction, and barrier dysfunction may contribute to malabsorption, increased epithelial permeability, and enhanced dissemination from the intestinal barrier, consistent with mild clinical manifestations observed during NPEV-associated disease.

### Innate immune responses

While epithelial tropism determines the initial sites of infection, the extent to which NPEVs disseminate beyond the intestinal barrier is likely influenced by immune responses. Understanding regional and serotype-specific differences in antiviral immunity is, therefore, critical for defining NPEV pathogenesis. Although antiviral responses are broadly conserved across NPEVs, variation exists in magnitude and composition. In fetal intestinal organoids, E11 triggers a more robust antiviral and pro-inflammatory cytokine response than CVB3 and EV-A17 (124). A comparison between EV-A71 and CVA16 in enteroids and colonoids demonstrated a stronger induction of IFNs, IFN-stimulated genes (ISGs), and pro-inflammatory cytokines by EV-A71 (126). Serotypes also differ in their susceptibility to IFN-mediated restriction: EV-A71 is primarily restricted by IFN-λ3, whereas CVA16 is restricted by both IFN-λ1 and IFN-λ3 (126). The regional distribution of IFN responses along the intestinal tract adds another layer of complexity. In human small intestinal epithelial cells, EV-D94 and EV-D68 induce low levels of type-I- and type-III-IFNs and ISGs (133). In mouse and fetal enteroids, EV-A71 and E5 predominantly induced type-III-IFN responses (IFN-λ2/3), accompanied by modest induction of type-I- and type-II-IFN. In addition to type-III-IFN, EV-A71 also upregulated the pro-inflammatory chemokine CXCL-10 (128, 134). Given that type-III-IFN acts locally at epithelial barriers to restrict viral replication while minimizing systemic inflammation, antiviral defense in the small intestine appears spatially contained. In contrast, EV-A71 infection of colonoids induces substantial type-I- and type-II-IFN responses alongside type-III-IFN, indicating that infection in the colon may trigger a broader immune activation and increased recruitment of inflammatory cells.

### Viral egress and dissemination

An unresolved question in NPEV pathogenesis is how NPEVs exit infected intestinal cells and disseminates beyond the gut. Emerging evidence points to multiple egress strategies, each with important implications for immune evasion and systemic spread (135, 136).

#### Extracellular vesicle-mediated release

NPEVs can exit host cells in extracellular vesicles through non-lytic release in different cell types (137, 138). In those, virions are encapsulated within extracellular vesicles and released without destroying the infected host cell. In large intestinal epithelial cells, EV-A71 strain MP4 increased exosome production and released virions within these vesicles (131). By hijacking this pathway, viral dissemination may occur while evading neutralizing antibodies.

#### Whole-cell extrusion

In differentiated colon organoids, EV-A71 strain 4643 induced displacement of intact infected cells from organoids into the extracellular environment, facilitating spread to neighboring cells (129). Extruded cells lacked apoptotic features, suggesting that apoptosis is not the primary driver of this process. Instead, extrusion of infected cells appears to depend on the force-sensing activity of mechanosensitive ion channels such as Piezo1, as inhibition with GsMTx4 reduced cell extrusion (129). Similar observations have been made for poliovirus, suggesting that extrusions of infected cells may represent a conserved dissemination strategy among NPEVs adapted to the GI-tract (129).

#### Upstream open reading frame

The recently discovered upstream open reading frame (uORF) in the EV genome encodes a membrane-associated protein that acts as a membrane-disrupting factor required for the release of virus from within vesicles. Intestinal organoids were instrumental in identifying this pathway because uORF is essential for replication in organoid systems yet dispensable in cell lines for fecal–oral transmissible NPEVs (139). uORF-knockout viruses showed reduced titers, decreased virus titer/protein and virus titer/RNA ratios at late infection stages, and increased the proportion of membrane-associated virus, collectively underscoring the role of the uORF-encoded protein in facilitating vesicular virus release during gut epithelial cell infection. Comparative genomic analysis revealed broad conservation of the uORF among *Enterovirus alphacoxsackie* and *betacoxsackie* species and approximately half of *Enterovirus coxsackiepol* isolates, whereas it is entirely absent in *Enterovirus deconjuncti* species, including EV-D68 (139). Consistent with this, EV-D68 generally replicates poorly in intestinal organoids. However, EV-D68 isolate KY/14/18953 replicated to high levels in human enteroids; the reason for the high replication capacity is unknown (140). This isolate contains amino acid substitutions in capsid protein VP1, which is involved in receptor recognition, suggesting that receptor usage may also be important in determining intestinal replication capacity.

Collectively, intestinal organoid systems have significantly improved our understanding of early NPEV pathogenesis. These models demonstrate that infection outcomes are shaped by interactions between receptor usage, epithelial polarity, intestinal region, host-cell differentiation status, and viral genotype, which together influence tropism, epithelial damage, innate immune responses, and viral egress. The predominance of type-III-IFN responses in the small intestine, compared with the broader type-I-, type-II-, and type-III-IFN responses observed in the colon, suggests regional differences in antiviral defense and susceptibility to dissemination, possibly representing a gateway for systemic spread. Additionally, NPEVs employ multiple non-lytic dissemination strategies that facilitate viral spread while preserving epithelial integrity and potentially avoiding immune recognition. Despite these advances, key questions remain regarding the receptor repertoire governing epithelial entry, the mechanisms driving cell death in differentiated intestinal cells, and how localized infection progresses to systemic disease.

## RESPIRATORY SYSTEM

Although respiratory disease is most associated with EV-D68, other NPEV serotypes can cause disease in the airways (Table 1). Despite the clinical importance, the target cell types and mechanisms underlying respiratory pathogenesis remain poorly understood.

### Respiratory cell tropism

Airway epithelium cultures differentiated at ALI including adult nasal epithelium as well as fetal and pediatric tracheal/bronchial epithelium support replication of EV-D68, CVA13, CVA20, EV-C99, and CVB4 (125, 141, 142). In all cultures, ciliated airway epithelial cells are the predominant target of infection indicating shared cellular tropism among these NPEVs despite variations in replication kinetics.

### Determinants of respiratory pathogenesis

Comparison among serotypes demonstrates substantial variations in replication and cytopathogenicity. Within the species *Enterovirus deconjuncti*, EV-D68 and EV-D94 display distinct replication phenotypes, underscoring differences between respiratory- and fecal-oral-transmitted NPEVs. Increased EV-D68 replication leads to higher cytotoxicity, loss of ciliated cells, and reduced mucociliary clearance. Evidence from chimeric viruses suggests that capsid-encoded determinants are major drivers of respiratory cytopathogenicity (143). These findings identify ciliated airway epithelial cells as a common target of NPEVs while highlighting substantial differences in their capacity to replicate and damage the epithelium.

Beyond serotype-level differences, replication kinetics can vary considerably among strains. Studies using airway organoids infected with EV-A71 strains from clades B3, B4, C1, C2, C4, and C5 identified a polymorphism at amino acid position 145 of the VP1 protein as a key determinant of replication efficiency (144). Strains harboring glutamine at VP1 position 145 (VP1-145Q) replicated more efficiently than viruses encoding a glutamic acid or glycine (VP1-145E/G), a substitution that has been linked to heparan sulfate binding (145).

The clinical importance of strain-specific variation is illustrated by the two CVB4 GZ strains, with distinct disease origins. The CVB4 strain GZ-R6 isolated from a patient with pneumonia induces more pronounced epithelial damage characterized by loss of the tight-junction protein ZO-1, reduced α-tubulin levels indicative of ciliary loss, early disruption of epithelial morphology, and nuclear enlargement consistent with epithelial hypertrophy (142). In contrast, these pathological changes are less pronounced following infection with CVB4 strain GZ-HFM01, a stool-derived isolate obtained from a patient with HFMD and aseptic meningitis (142).

Host factors further modulate the outcome of respiratory NPEV infection, influenced by age and underlying comorbidities. Human airway epithelial cultures derived from fetal or adult tissue supported replication of CVA13, CVA20, and EV-C99 with serotype-dependent differences, but overall viral titers were higher in adult-derived than in fetal-derived cultures (125). Underlying respiratory diseases such as asthma and COPD modify infection outcomes. EV-D68 has been associated with asthma exacerbations (146), and human airway epithelia obtained from nasal or bronchial asthma patients’ tissues exhibit increased permissiveness to EV-D68. This enhanced permissiveness correlates with diminished mucociliary clearance, suggesting that pre-existing epithelial dysfunction may facilitate susceptibility to viral infection (119). Similar approaches offer opportunities to investigate the impact of other chronic respiratory diseases, including COPD.

Together, these findings demonstrate that respiratory pathogenicity is host-factor-, serotype-, and strain-dependent and that relatively minor genetic differences can influence viral replication and epithelial damage, likely contributing to differences in disease severity.

### Innate immune responses

The innate immune response to respiratory NPEV infection is both serotype- and strain-dependent. EV-D68 induced the strongest antiviral response in airway ALI models, characterized predominantly by type-III-IFN production irrespective of apical or basolateral infection, with apical infection triggering a more robust response (140). In small airway epithelial cultures, EV-D68 induced type-III-IFN alongside IFN-β (133). Notably, type-I- and type-II-IFN induction were absent, suggesting that the antiviral defense against EV-D68 in the airway epithelium is spatially contained, analogous to the observation in the small intestine (133, 140). Contemporary EV-D68 strains induce broadly similar transcriptional responses, encompassing upregulation of OASL, DDX60, IFNλ1-3, CXCL10, and CXCL11 (147). However, strain-dependent differences remain apparent. Contemporary strains induce stronger antiviral and pro-inflammatory cytokine profiles than the laboratory-adapted EV-D68 Fermon strain, which shows both reduced replication and diminished antiviral responses. Other NPEV serotypes trigger little or no innate immune pathways; EV-D94 was unable to induce antiviral pathways and instead suppressed innate and inflammatory responses, an effect that is not rescued by the addition of THP-1 macrophages in respiratory organoids (133). In the immortalized cell line 16HBE derived from bronchial epithelium EV-A71 and CVA16 infection failed to induce innate immune responses (148).

### Viral egress and dissemination

In polarized Calu-3 transwell cultures and multicellular ALI models, EV-D68 was released from the apical site following infection. Nevertheless, low-level basolateral release could not be excluded following either apical or basolateral infection. Basolateral virus detection was associated with disruption of transepithelial electrical resistance and tight junction integrity, suggesting that epithelial barrier breakdown may facilitate dissemination beyond the airway lumen (125, 140). EV-C99 represents an exception among NPEVs, as basolateral release was detected (125). These findings suggest that directional viral shedding towards luminal surfaces may be a conserved feature of NPEV infection across respiratory and GI epithelium, whereas basolateral release may provide a potential route for dissemination.

Advanced airway organoids and ALI models have substantially improved our understanding of respiratory NPEV pathogenesis. These studies identified ciliated airway epithelial cells as the primary targets and show that viral genotype, host conditions, and infection polarity collectively shape replication efficiency, cytotoxicity, and epithelial damage. EV-D68 induces a type-III-IFN response, whereas most fecal-oral transmitted NPEVs trigger little innate immune activation in respiratory models. Despite recent advances, the determinants of respiratory tropism, the significance of basolateral release in systemic dissemination, and the contribution of cytotoxicity and host immune responses to disease remain unclear. Notably, although upper respiratory tract-derived organoids have been applied to study rhinovirus infection (149), comparable studies of NPEVs are lacking.

## CENTRAL NERVOUS SYSTEM

Neurological complications are among the most severe outcomes of NPEV infections. Although only a minority of infected individuals develop neurological sequelae, NPEVs can infect the spinal cord, brainstem, and meninges, as well as peripheral nerves, causing encephalitis, meningitis, and AFP (10). Despite the clinical significance, the mechanisms governing NPEV neurotropism (the ability of a virus to infect and replicate within neuronal cells) remain incompletely understood.

### Neurotropism

Region-specific lesions between serotypes have been observed: EV-A71 affects the brainstem and spinal cord, with lesions in the ventral, medial, and caudal areas of the medulla oblongata, whereas CVB3 has been associated with lesions in the cortex and hippocampus (150, 151). Autopsies further suggest that NPEVs infect various CNS cell types, including neuron progenitor cells, glial cells, and neurons, resulting in neurodegeneration and local inflammatory responses (152). Consistent with these observations, CVB3 infects neural progenitor cells in both primary cell cultures and animal models, causing neurodegeneration in the subventricular zone and hippocampus although the relevance of these findings to human disease remains unclear (153, 154). Studies using hPSC-derived neural cultures further demonstrate serotype-specific differences in cellular neurotropism. In co-cultures of hPSC-derived cortical neurons and astrocytes, as well as in primary rat cortical neural cultures, EV-D68 predominantly infects neurons with some infection of astrocytes, while EV-A71 infects both neurons and astrocytes, although astrocytes have not been evaluated in primary rat cultures (155, 156). In spinal cord/hindbrain assembloids, EV-D68 preferentially infected motor neurons with limited astrocyte tropism, while EV-A71 showed tropism for both cell types (157). These findings suggest that CNS disease results from a combination of serotype-specific neurotropism and differential susceptibility of neural cell populations, which may partly explain the distinct neurological manifestations.

### Determinants of neurotropism

The molecular determinants governing NPEVs’ neurotropism remain poorly defined. The apparent receptor promiscuity of NPEVs suggests that neuroinvasion is unlikely dependent on a single receptor (Table 2). Sialic acid (SIA) expression appears to influence EV-D68 susceptibility. In forebrain organoids, early developmental stages express higher levels of SIA than later stages. Infection studies using SIA-dependent and SIA-independent EV-D68 strains demonstrated that the MO strain infected only early-stage organoids, whereas both MO and Fermon strains infected late-stage organoids (158). Neuraminidase treatment reduced viral RNA levels in a strain- and developmental stage-dependent manner but reduced infectious virus production only for the MO strain, indicating that SIA alone cannot explain differences in neurotropism (158, 159). EV-D68 strains B2/2042, B2/947, and A1/1348 infect cerebral organoids, with B2/2042 showing dependence on SIA but not on heparan for infection (141). The recently identified MFSD6 slightly reduced viral titer when knocked out, suggesting that MFSD6 may only partially contribute to neurotropism (54). Together, these findings indicate that neurotropism depends on serotype- and context-dependent attachment factors rather than a single dominant receptor.

### Neuroinvasion

The frequency and mechanism of neuroinvasion remain poorly understood. For poliovirus, paralytic disease occurs in approximately 1 in 200–1,000 poliovirus infections whether a comparable ratio applies to NPEVs remains unknown (160). NPEVs can enter the CNS through two routes: hematogenous spread and transaxonal transport.

#### Hematogenous spread

During viremia, circulating NPEV particles may enter the CNS by crossing the blood-brain barrier (BBB) or the blood-cerebrospinal fluid barrier (BCSFB). Alternatively, infected immune cells may transport the virus across these barriers via so-called Trojan-horse mechanisms.

##### Blood-brain barrier

The BBB is formed by brain microvascular endothelial cells connected by tight junctions and supported by pericytes and astrocytes (Fig. 2C). Disruption of neurovascular integrity facilitates viral neuroinvasion through both direct infection of endothelial cells and/or inflammatory-mediated alterations in barrier permeability (161). Consistently, infection of cerebral microvascular endothelial cell lines and primary brain microvascular endothelial cells disrupts the endothelial cell barrier *in vitro (*162). CVB3 infection activates TLR-8 signaling in brain microvascular endothelial cells mounting a type-I-IFN response that may decrease BBB permeability (163). Serotype-dependent differences have been observed in a human BBB co-culture model consisting of brain-like endothelial cells and pericytes in which E6 replicated efficiently and disrupted barrier function, whereas EV-A71 replicated poorly and failed to infect pericytes (164). These studies indicate that some NPEV serotypes directly infect BBB-associated cells and compromise barrier integrity, while others may rely on indirect mechanisms, such as systemic cytokine-mediated disruption of endothelial junctions, thereby facilitating viral entry, although direct experimental evidence for this mechanism is currently lacking.

##### Blood-cerebrospinal fluid barrier

The BCSFB is formed by choroid plexus epithelial cells associated with endothelial cells and constitutes another potential route for neuroinvasion. Using human choroid plexus papilloma cells (HIBCPP) as a simplified BCSFB model (Fig. 2C), E30 was shown to infect epithelial cells in a strain-specific pattern resulting in loss of barrier integrity (165, 166). Infection also augmented secretion of the chemokines CXCL1, CXCL2, CXCL3, IL-8, and CCL5. Despite enhanced chemokine production, T-cell influx was not increased (162), suggesting that local chemokine signaling alone is insufficient to drive immune cell transmigration across the BCSFB.

##### Trojan-horse transport

The ability of NPEVs to use the Trojan-horse mechanisms may depend on strain-specific tropism for lymphocytes. EV-A71 infects leukocytes, including B-, NK-, and T-cells promoting BBB transmigration via PSGL-1-mediated binding and caveolin-dependent endocytosis (164) (Table 2). In EV-A71, the amino acid at position 145 of VP1 determines binding to PSGL-1 on Jurkat T cells. Replication efficiency in lymphocytes varies considerably across EV-A71 strains and cell types: C1-06 RNA levels were comparable between T- and B-cells; C1-16 RNA was slightly higher in B-cells, and C4 RNA levels were uniformly low across both lymphocyte populations (164). Also EV-D68 strains showed strain-dependent immune cell tropism. Clades A and B2 infected B-cells, whereas clade A2 did not. Neuraminidase treatment reduced infection across all strains, implicating SIA-binding affinity as a contributing factor for B-cell entry (167). Additionally, dendritic cells (DCs) are susceptible to NPEV infection: EV-D68 infects both immature and mature DCs with higher susceptibility in immature cells (167). EV-A71 strains can also infect DCs; infection is efficiently blocked by soluble heparin, suggesting a role for sGAGs in viral entry (Table 2) (168). However, the receptor repertoire governing NPEV infection of immune cells remains incompletely understood. In addition to infected leukocytes, microvesicles and extracellular vesicles have been implicated in EV-A71 transmigration across the BBB *in vitro* (169, 170).

### Transaxonal transport

Transaxonal transport via peripheral and cranial nerves is an established neuroinvasion route supported by animal studies (171, 172). It is hypothesized that NPEVs traverse the neuromuscular junction and exploit retrograde transport to invade the spinal cord and brain from the periphery, analogous to poliovirus. Supporting this model, pathological examination of an EV-D68 case showed virus antigen and viral RNA within motor neurons in the anterior horn of the spinal cord (173). Direct evidence for retrograde transaxonal transport has been obtained using microfluidic chambers containing hPSC-derived motor neurons. Infection of distal axons with EV-D68 resulted in the detection of viral antigen within neural cell bodies, and transport was reduced by nocodazole, confirming a microtubule-dependent mechanism (174).

### Neuropathogenesis

Infection with EV-D68 and EV-A71 impairs synaptic plasticity and synchronous neural activity in both hPSC-derived neural co-cultures and primary rat cortical neurons (155, 156). Whether NPEV infection impairs neural activity in the infected brain organoids remains to be established. Neural dysfunction may partially arise from cell death. In hPSC-derived cortical neuron-astrocyte co-cultures, EV-A71 induced the apoptosis, whereas EV-D68 predominantly triggers non-apoptotic cell death (156). In motor neurons, EV-A71 induces ferroptosis (175), while E11 triggers apoptosis in spinal cord organoids (176). The molecular mechanisms underlying EV-D68-associated neurodegeneration remain to be elucidated. Together, these observations indicate that NPEV-induced neurological injury encompasses both functional disturbances of neuronal networks and serotype-specific cell death pathways.

Although NPEV-induced neuronal injury is increasingly recognized, how it leads to motor dysfunction remains unclear. Increasing evidence supports retrograde neuronal transport as a mechanism underlying AFP. One unresolved question is whether muscle weakness primarily results from direct infection of skeletal muscle, degeneration of motor neurons, disruption of neuromuscular junction function, and thus loss of skeletal muscle innervation or a combination of these processes. Recent advances using hPSC-derived skeletal muscle myotubes (Fig. 2C) demonstrated that EV-D68 infection causes cellular damage and impairment of 3D myotube architecture (177). Likewise, hPSC-derived spinal motor neurons and spinal cord organoids are susceptible to NPEV infection, with replication kinetics varying among serotypes, indicating virus-dependent differences in viral growth dynamics across models (176). However, these systems cannot determine the direction of viral movement across the neuromuscular junction. More recently, assembloids combining human spinal cord organoids (hSpO) with human skeletal muscle organoids (hSkM) provided additional insight (Fig. 2C). Upon EV-D68 and EV-A71 infection, muscle tonus decreased, while viral antigen was detected primarily in hSpO, and only sporadically within the hSkM (157). Inhibition of viral replication with the 3C^pro^ inhibitor rupintrivir prevented loss of contraction, suggesting that active viral replication, rather than immune-mediated effects, directly mediates contraction impairment (157). Together, these data support a model in which NPEVs spread haematogenously to peripheral tissue and subsequently access the spinal cord through retrograde axonal transport. Nevertheless, the sudden onset of paralysis and the limited detection of viral antigen in muscle tissue suggest that disturbances in neuromuscular transmission and denervation may contribute more substantially to weakness than direct muscle destruction alone (178).

### Neuroinflammation/neurovirulence

In addition to the virus-induced changes in neural activity, local neuroinflammatory responses may contribute substantially to disease severity. Histopathology studies showed that EV-D68 infection of motor neurons in the spinal cord is accompanied by an influx of immune cells, potentially contributing to dysregulation of neural homeostasis (173, 179). Despite the absence of immune cells in hSpOs and cortical neuron–astrocyte co-cultures, NPEV infection elevated proinflammatory cytokines such as IL-6, IL-8, and CCL5, and induced minimal type-I- and type-III-IFN responses (156, 157). These factors may facilitate the recruitment of different lymphocyte cells to the sites of infection. IFN signaling appears to exert strain-dependent antiviral effects: blocking IFN signaling with JAK1/2 inhibitor ruxolitinib increased RNA levels of EV-D68 strain Fermon and MO, but not MD (158). Pretreatment of neuron progenitor cells with type-I-IFNs suppressed CVB3 replication, whereas IFN-γ enhanced it (153), raising the possibility that IFN-γ may exert pro-viral effects in the CNS. Whether such responses contribute directly to the neuropathogenesis remains unclear.

Taken together, current evidence supports a multistep model of NPEV neuropathogenesis in which viruses access the CNS through hematogenous dissemination and/or retrograde axonal transport, infect distinct neural cell populations, and induce pathology through a combination of neuronal dysfunction, cell death, and neuroinflammation. Neurons are a major target across serotypes although the susceptibilities of astrocytes, neural progenitor cells, and immune cells vary between viruses. Despite considerable advances, several key questions remain unresolved, including the relative importance of different neuroinvasion routes in humans, the mechanisms responsible for AFP, the contribution of direct viral injury vs immune-mediated damage, and the receptor determinants underlying serotype-specific neuropathogenesis.

## CARDIAC SYSTEM

CVBs and echoviruses are frequently associated with cardiac complications, including myocarditis, pericarditis, and myopericarditis (Table 1). CVBs are responsible for approximately 25%–40% of myocarditis and cardiomyopathy cases in children and young adults (180). Histopathological examination revealed edema, lymphocyte and neutrophil infiltration, and cardiomyocyte necrosis (181). Although myocardial biopsies remain uncommon, CVB3 antigen has been detected in cardiomyocytes (182, 183).

### Cardiac tropism and pathology

iCell cardiomyocytes are permissive to CVB3 infection, demonstrated by cytoplasmic staining confirming the presence of both CVB3 RNA and antigen (184). The morphological changes observed in this *in vitro* model closely recapitulated those previously described in the well-established *in vivo* murine model of CVB3 infection (184). The formation of replication organelles confirmed active viral replication within infected cells (184). Infected cardiomyocytes did not undergo apoptosis, unlike respiratory and GI cells, but instead underwent necroptosis. Cardiomyocyte necroptosis was measured by the activation of receptor-interacting protein kinase 1 (RIPK1) and receptor-interacting protein kinase 3 (RIPK3), accompanied by loss of membrane integrity, myofibril disruption, and mitochondrial alterations (184). To determine whether these kinases were essential for CVB3 replication, infected cells were pre-treated with GSK'872 (RIPK3 inhibitor) or necrostatin (RIPK1 inhibitor). These inhibitors significantly attenuated viral replication, indicating that the activity of both kinases is required for CVB3 replication (184). Furthermore, mitochondrial alterations may suggest metabolic dysregulation in infected cardiomyocytes; whether these changes are directly virus-induced and whether they contribute to the overall pathology of cardiac disease remains to be elucidated.

### Innate immune responses

While direct infection of cardiomyocytes by NPEVs initiates cell death, the immune system activation can also influence cardiac pathology. A study utilizing AC16 cardiomyocytes identified significant transcriptional reprogramming by E6 and CVB5, with differentially expressed genes predominantly associated with immune response and antiviral defense mechanisms (185). The distinct enrichment profiles observed between CVB5 and E6 suggest that virus-specific replication strategies and differential host factor interactions drive immune activation and signaling responses. Both serotypes induced expression of IFN-β, IFN-λ1, and IFN-λ3. The type-I-IFN induction is relevant given its pro-inflammatory nature in cardiomyocytes, in contrast with the predominantly type-III-IFN-driven responses characteristic of respiratory and enteric infection models, suggesting tissue-specific divergence in antiviral strategy. Infected cardiomyocytes produce additional cytokines and chemokines, suggesting a broader innate immune and inflammatory response upon NPEV infection (185). Clinical evidence from CVB3-diagnosed patients complements these *in vitro* observations: CVB3-induced myocarditis showed upregulation of regulatory microRNAs (miRNAs; miR-146b and miRNA-214) in heart tissues. These miRNAs target ITCH, a suppressor of NF-kB, thereby increasing TNF-α and IL-6 production, potentially contributing to myocardial dysregulation (186). Data on immune induction in co-culture systems combining cardiomyocytes with immune cells are absent from the literature, representing a significant knowledge gap in fully understanding the pathogenesis of NPEV-associated cardiac disease (185).

## SKIN AND MUCOSAL SURFACES

### Skin pathogenesis

NPEVs can cause HFMD, a transient maculopapular **r**ash involving the mouth and frontal area, spreading rapidly to the whole face, extremities, hands, and feet, including palms and soles. Histology of CVA6-induced HFMD demonstrates acute epidermal injury with intraepidermal vesiculation due to spongiosis and ballooning degeneration, progressing to reticular degeneration, keratinocyte apoptosis, and superficial epidermal necrosis with neutrophilic sloughing and perivascular lymphocyte infiltrates (181). Similar to pathological findings, EV-A71 infection of skin organoids resulted in infection of multiple cell types, including keratinocytes, epidermal cells, and nerve cells (181, 187). In organotypic human skin and mucosal cultures derived from prepuce and lip biopsies, as well as in primary keratinocyte cultures, EV-A71 viral RNA and antigen were detected in keratinocytes and mucosal squamous epithelial cells (187). In prepuce and lip skin organotypic cultures, infected squamous cells were predominantly localized beneath the stratum corneum, while in oral mucosa cultures, infected cells were additionally observed within the most superficial epithelial layers (187). These findings indicate that keratinocytes are the target cells for viral replication, contributing to cutaneous viral shedding, as evidenced by the release of virus particles, which may contribute to person-to-person transmission (187).

### Mucosal pathogenesis

Acute hemorrhagic conjunctivitis (AHC) is a highly contagious disease associated with NPEV infection of the superficial cells of the conjunctiva and cornea (Table 1). To assess subconjunctival membrane susceptibility to NPEV, human umbilical vascular endothelial cells (HUVECs), human corneal epithelial cells (HCEs), and primary conjunctival cells were used (88, 188). HCEs and HUVECs show cytopathic effect following EV-D70 infection (188). EV-D70 infection triggers differential temporal kinetics of ICAM-1 expression in HCEs and HUVECs, highlighting distinct cell type-specific mechanisms that may facilitate leukocyte infiltration and ocular inflammation (188). Studies utilizing normal human conjunctival (NHC) and HCE cells suggest that CVA24v engages distinct receptors in each cell type (189). Inhibition of O-glycans significantly impaired infection in HCE cells compared to NHC cells, indicating a differential receptor dependency between these two ocular epithelial cell types (189). Local immune responses are postulated to increase vascular permeability by damaging small capillaries, potentially contributing to disease pathogenesis; however, the precise underlying mechanisms remain to be elucidated.

## OUTLOOK FOR THERAPEUTIC STRATEGIES USING ADVANCED CELL MODEL SYSTEMS

No antiviral treatment options exist for NPEV infection. Direct-acting antivirals such as capsid binders (e.g., Pleconaril, Pirodavir, Vapendavir, Pocapavir), protease inhibitors (e.g., Rupintrivir [AG-7088], its analog AG7404 [V-7404]), or host-targeted antivirals (Enviroxime) have been halted or failed in clinical trials due to poor efficacy or toxicity issues (190–192). The limited commercial market for anti-enteroviral therapeutics discourages industrial investment in developing broad-spectrum anti-enterovirals. However, there is a great need for such, as infection can lead to life-threatening and sometimes even fatal complications. Besides this, antivirals are also essential tools for achieving polio eradication and managing the post-eradication period (193).

A promising target for direct-acting antiviral drug development is the highly conserved non-structural protein 2C (194). The FDA-approved drug fluoxetine, clinically used as an antidepressant, has been identified as an allosteric inhibitor of the species *Enterovirus betacoxsackie* and *deconjuncti* (195, 196). Fluoxetine has one chiral center, and while both enantiomers are active on the serotonin reuptake, the antiviral activity is executed by the *S*-enantiomer (195, 197). Clinically, fluoxetine has been used off-label in immunocompromised patients with severe NPEV infection, with reported clinical improvement depending on the timing of administration and the specific viral serotype involved (198–202). Optimization of fluoxetine derivatives to enhance the spectrum of antiviral activity while eliminating the neurotoxic adverse effect can be a promising approach (203). Conventional cell lines are suitable for antiviral screenings; however, advanced cell models such as hPSC-derived neural cultures are desirable for evaluating antiviral efficacy alongside potential neurotoxicity (156, 203). Importantly, optimization of fluoxetine-based compounds has demonstrated that fluoxetine-based inhibitors with low nanomolar potency against NPEVs can be generated, highlighting the therapeutic potential of this compound class (203–205).

Host-directed antivirals targeting the phosphatidylinositol 4-phosphate (PI4P)–PI4KB–OSBP axis which is critical for the replication of NPEVs have been developed (206). Inhibitors such as enviroxime and related PI4KB-targeting compounds have demonstrated broad-spectrum antiviral activity in cell lines (207). However, clinical development of enviroxime was halted due to toxicity issues and negative effects on lymphocyte function in *in vivo* models (208). This shows that more advanced cell models are needed for preclinical development of drugs targeting essential proteins (209). Especially multicellular models and organoids supplemented with immune cells can help in identifying potential off-target effects such as disruption in lymphocyte function. Currently, a new class of PI4KB inhibitor is undergoing clinical evaluation for cancer treatment with improved safety profiles (210); whether these inhibitors are suitable for anti-enteroviral therapy needs to be established.

Advanced patient-derived systems also support personalized antiviral approaches, especially in rare or severe manifestations of NPEV infections (211). Testing of repurposed drugs in physiologically relevant models can provide an accurate prediction of therapeutic efficacy compared to conventional cell lines. A recent case exemplified this principle: although remdesivir showed antiviral activity against CVA1 in standard cell-based assays, it failed to clear infection both in the patient and in human fetal intestinal epithelium organoids (211). In addition to antivirals, other therapeutic modalities, such as virus-specific monoclonal antibodies, can be systematically evaluated in advanced models (212). Such platforms enable assessment of antiviral potency, tissue-specific efficacy, and potential off-target effects, thereby supporting rational selection of individualized therapeutic strategies.

## FUTURE PERSPECTIVES AND CONCLUSIONS

Recent NPEV outbreaks and their association with severe neurological complications among other manifestations have highlighted the need for a better fundamental understanding of their pathogenesis. The rapidly evolving field of advanced cell models has provided valuable tools to investigate viral tropism, replication dynamics, host immune responses, and serotype-specific phenotypes; nevertheless, limitations remain. *In vitro*-derived cell types often exhibit molecular profiles that differ from their native tissue counterparts; current systems inadequately represent key immune cell populations and generally lack the longevity required to model prolonged or chronic infection. Moving forward, the key challenge is not only to refine individual models but also to integrate multiple physiological compartments. Because NPEV pathogenesis spans multiple systems, next-generation multi-organ systems incorporating functional immune components will be necessary to better investigate disease mechanisms.

On the clinical side, advances in standardization of experimental cell systems may have direct implications, particularly given the absence of approved antivirals. Patient-derived organoids could support a personalized treatment by assessing viral susceptibility to potential off-label treatment with FDA/EMA-approved drugs such as fluoxetine. In parallel, complementary strategies such as improving epidemiological surveillance help contextualize and validate findings from *in vitro* findings.

Ultimately, no single model system, human or animal, *in vitro* or *in vivo*, will be sufficient on its own. Continued progress will require the integration of physiologically relevant *in vitro* systems, well-validated animal models, and robust surveillance efforts to achieve a mechanistic understanding of NPEV pathogenesis and accelerate the development of antivirals, monoclonal antibodies, and vaccines.
